# Naphthalene Toxicity: Methemoglobinemia and Acute Intravascular Hemolysis

**DOI:** 10.7759/cureus.3147

**Published:** 2018-08-15

**Authors:** Giselle Volney, Michael Tatusov, Andy C Yen, Nune Karamyan

**Affiliations:** 1 Internal Medicine, Ross University School of Medicine, Roseau, DMA; 2 Medicine/Trauma and Critical Care, University of Maryland School of Medicine, Baltimore, USA; 3 Internal Medicine, Ross University School of Medicine, Baltimore, USA; 4 Internal Medicine, University of Maryland Prince George's Hospital Center, Cheverly, USA

**Keywords:** naphthalene, hemolysis, kidney injury, methemoglobinemia, ascorbic acid, toxicity, moth balls

## Abstract

Naphthalene poisoning is a rare form of toxicity that may occur after ingestion, inhalation, or dermal exposure to naphthalene-containing compounds such as mothballs. Clinically, patients present with acute onset of dark brown urine, watery diarrhea, and non-bloody bilious vomiting 48-96 hours after exposure. Vital sign abnormalities include fever, tachycardia, hypotension, and persistent pulse oximetry readings of 84%-85% despite oxygen supplementation. Laboratory workup demonstrates hyperbilirubinemia with indirect predominance, hemolytic anemia, methemoglobinemia, and renal dysfunction. Treatment options include supportive care, red cell transfusion, ascorbic acid, methylene blue, and N-acetylcysteine. We present a case of naphthalene toxicity in a 20-year-old autistic male, who improved with supportive care, red blood cell transfusion, and ascorbic acid.

## Introduction

Naphthalene, an ingredient in mothballs, is well absorbed following ingestion, dermal contact, or inhalation. Exposure often causes headache, altered mental status, vomiting, diarrhea, abdominal pain, and fever [1]. Ingestion of naphthalene mothballs also commonly causes hemolytic anemia and methemoglobinemia [2]. The acute intravascular hemolysis following naphthalene ingestion can lead to anemia, hematuria, leukocytosis with neutrophil predominance, jaundice, hepatic and renal dysfunction. Ingestion of as little as two naphthalene-containing mothballs can result in severe hemolytic anemia and hypotension. The toxic dose of naphthalene poisoning is not known [1]. However, ingestion of 12 mothballs has been reported, with resolution of hemolysis and methemoglobinemia after five days of aggressive treatment including adjunct continuous venovenous hemofiltration [3].

## Case presentation

A 20-year-old male patient with autism and attention deficit hyperactivity disorder was transferred to our facility from a local hospital, after presenting with sudden onset of dark brown urine, non-bloody bilious vomiting, and painless watery diarrhea two days earlier. History was obtained from the patient's mother, as the patient was mostly nonverbal. His mother reported subjected fevers, but denied chest pain, shortness of breath, recent travels, or sick contacts.

On presentation to the local hospital, the patient was febrile (100.8 F), tachycardic (heart rate 111 and regular), and had an elevated blood pressure (148/87). Pulse oximetry was 84%-85% on 100% non-rebreather mask. Venous blood gas at that time showed pH 7.55, partial pressure of carbon dioxide (pCO2) 21, and partial pressure of oxygen (pO2) of 27, although the patient was lying comfortably in bed with a respiratory rate of 16. The other measurements were as follows: hemoglobin 11 g/dL, blood urea nitrogen 18 mg/dL, creatinine 0.3 mg/dL, total bilirubin 12 mg/dL, amylase 95 U/L, lipase 47 U/L, and lactic acid 2.7 mmol/L.

The patient was transferred to our hospital for admission to the critical care unit. Within hours of presentation, the patient became febrile with a maximum temperature of 100.7 F. On physical examination, the patient's sclera was icteric, hands were pale and jaundiced, and his lower lip was also jaundiced. His heart sounds were regular, abdomen was soft, non-tender and non-distended to palpation, with normoactive bowel sounds and no rebound, guarding or hepatosplenomegaly. Arterial blood gas was consistent with respiratory alkalosis: pH of 7.49, pO2 of 201, pCO2 of 27, bicarbonate of 20.3 mmol/L, and a base excess of -2.4 mmol/L. Methemoglobin level was 14%. His labs included hemoglobin 6.9 g/dL and hematocrit 21.2. The patient had leukocytosis (22.2 with 64% neutrophils), a platelet count of 145,000, creatinine level of 0.4 mg/dL, and glomerular filtration rate >60. His total bilirubin was elevated at 11.6 mg/dL (normal range 0.2-1 mg/dL). Alkaline phosphatase (40 U/L) and alanine aminotransferase (ALT) (21 U/L) were within normal limits. On presentation, his direct and indirect bilirubin, and aspartate aminotransferase (AST) levels could not be obtained as blood samples frequently hemolyzed, but subsequent studies the following day showed total bilirubin of 8.0 mg/dL with indirect hyperbilirubinemia (7.1 mg/dL), AST 117 U/L (normal range 5-40 U/L), and ALT 24 U/L. His hepatitis panel was negative, prothrombin time was 16.6 seconds, international normalized ratio was 1.3, and partial prothrombin time was 34.0 seconds. Urine analysis showed brown urine with +3 blood, +2 protein, +1 ketones, and a microscopic examination showed only 1-5 red blood cells. Glucose-6-phosphate-dehydrogenase (G6PD) was 10.2 U/gHb (normal range 5.5-20 U/gHb). Magnetic resonance cholangiopancreatography showed a distended gallbladder with no large filling defects or gallstones, normal intrahepatic bile ducts and common bile duct with no evidence of biliary obstruction, and normal pancreas. The patient was initially treated with supportive care—intravenous normal saline.

**Figure 1:**
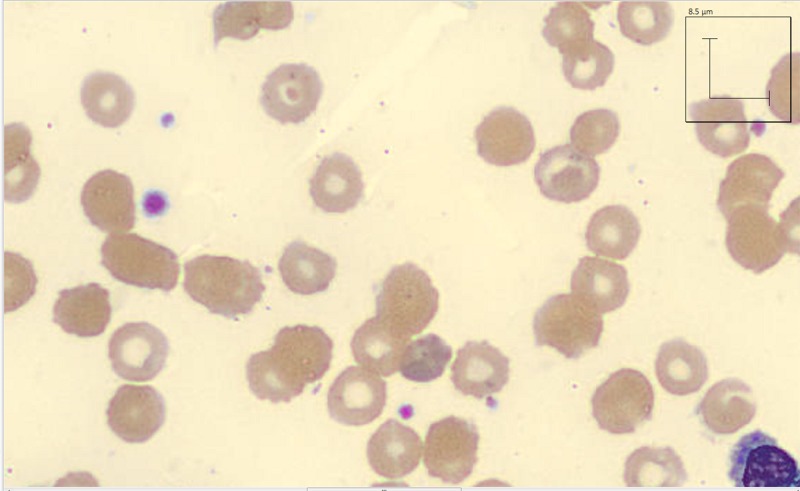
Peripheral smear showing red blood cell morphology of patient with naphthalene toxicity three days after admission.

**Figure 2:**
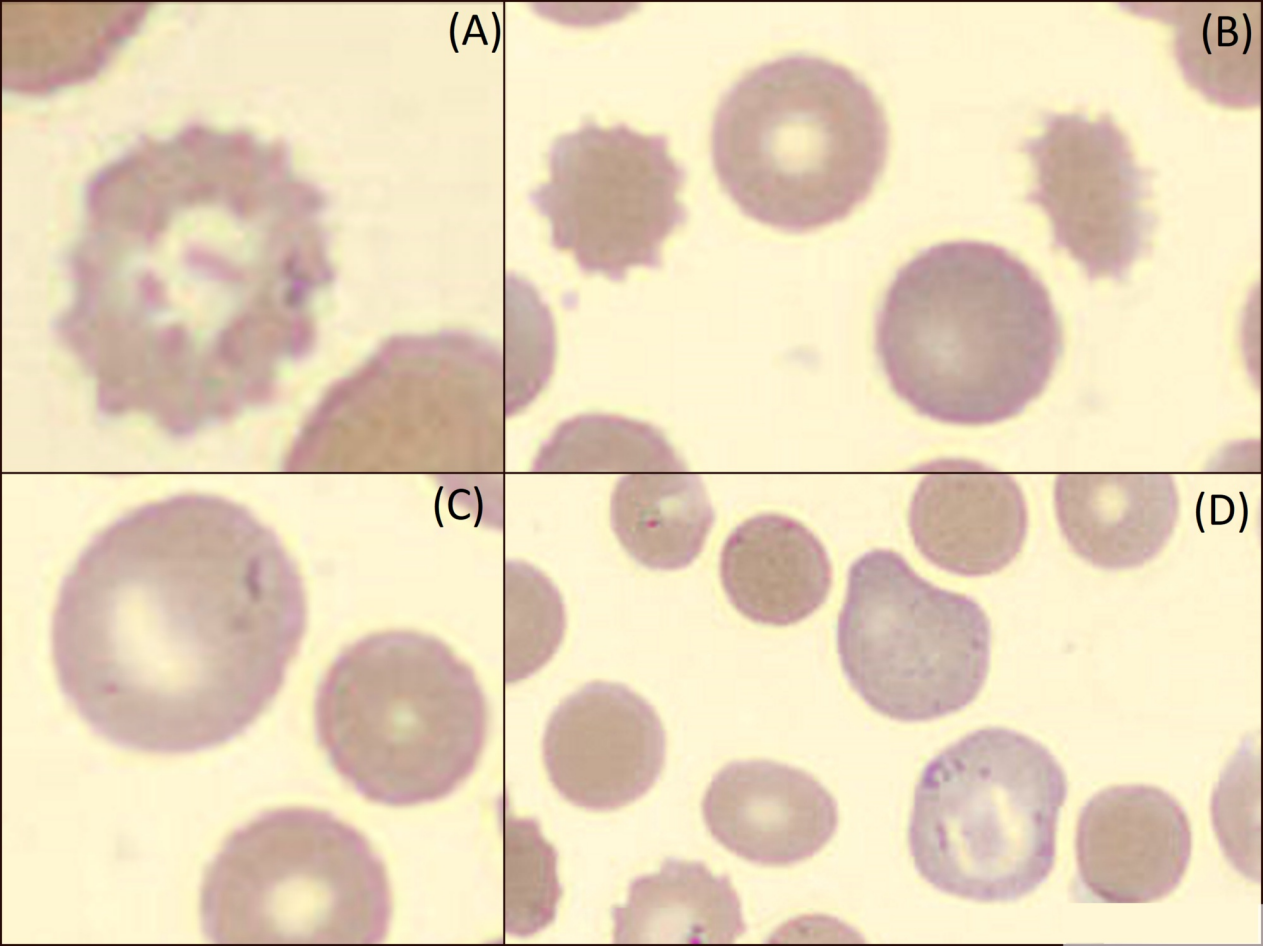
Burr cells (A, B), microcytes and macrocytes (B, C, D), and red blood cell morphology from peripheral smear following acute intravascular hemolysis secondary to naphthalene toxicity on Day 3 of hospital admission.

**Figure 3:**
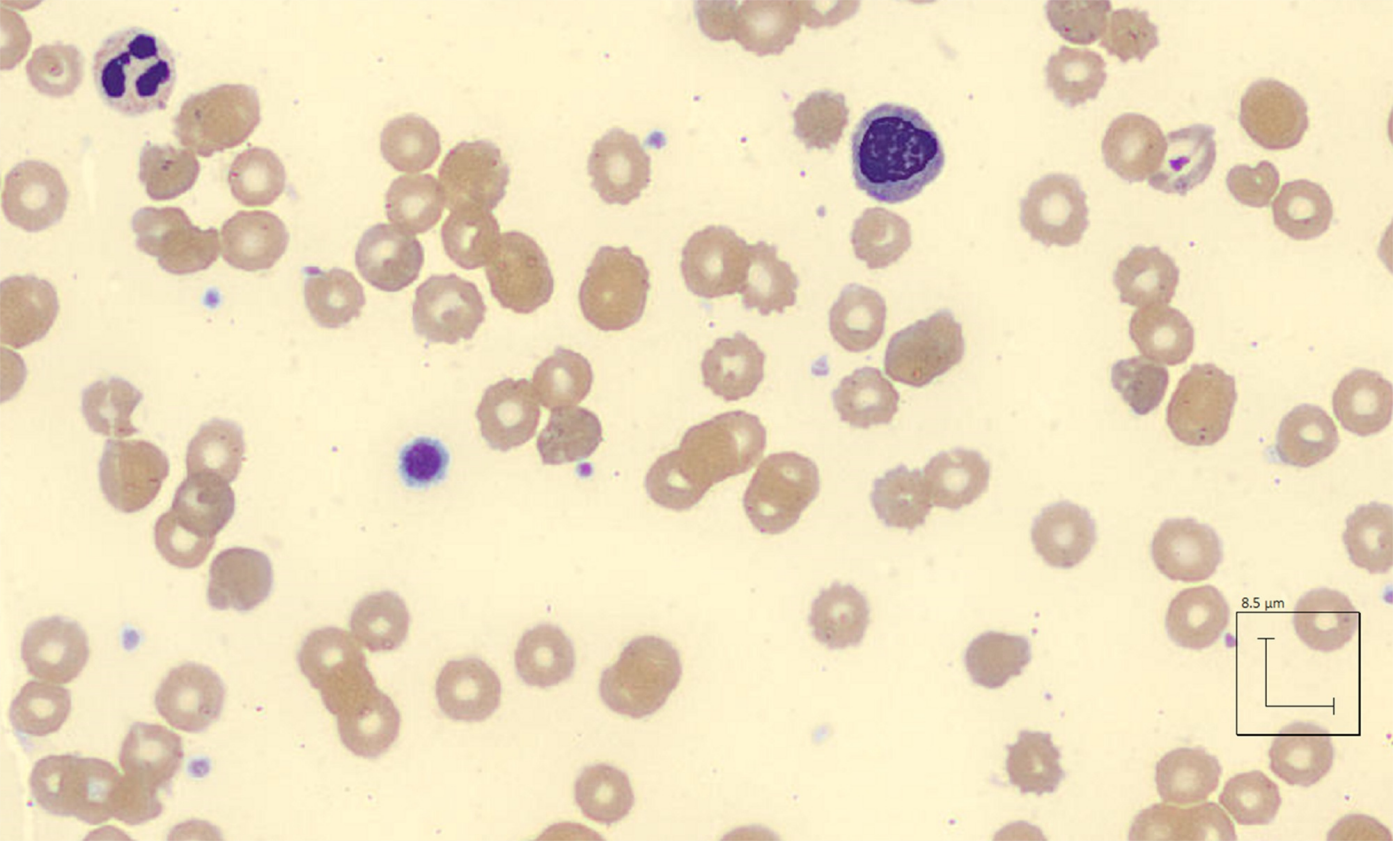
Microcytes, macrocytes, anisocytosis, poikilocytosis, bite cells, and Burr cells from peripheral smear following acute intravascular hemolysis secondary to naphthalene toxicity on Day 3 of hospital admission.

On Day 3 of admission, the patient was found unresponsive, diaphoretic, and tachycardic to 150 with oxygen saturation at 94% on room air. Hemoglobin was 3.6 g/dL, haptoglobin <10 mg/dL, reticulocyte count elevated 6.3%, and reticulocyte index 2.83. Lactate dehydrogenase was 2431 unit/L and total creatine kinase was 319 unit/L. Peripheral blood smear showed microcytes, macrocytes, Burr cells, Heinz bodies, bite cells, anisocytosis, and poikilocytosis (Figures 1-3). Creatinine was also noted at 1.4 mg/dL, suggestive of acute kidney injury. The methemoglobin level was 10%, with carboxyhemoglobin level at 4.4%.

The patient was transfused four units of packed red blood cells with appropriate response to 9.4 g/dL. Respiratory support was provided with 5 liters nasal cannula with response oxygen saturation to 97%. Upon further questioning, the mother admitted that the patient was exposed to naphthalene mothballs, which she recently laid down in her home. The mode and amount of exposure was not known.

The patient was treated with intravenous normal saline and ascorbic acid (225 mg) orally three times a day. The patient began clinically improving and did not require inotropic support, intubation, or hemodialysis. Methylene blue was considered but was not given to the patient as the patient was clinically improving and methemoglobin levels were trending down. The patient was not oliguric during the hospital stay and continued to urinate copious amounts of dark brown urine, which improved to an amber color on the day of discharge. His hemoglobin remained stable between 9-10 g/dL after four units of packed red blood cells for the rest of his hospital stay. The methemoglobin level trended down to 2% on the day of discharge. Creatinine and total bilirubin trended down to 0.8 mg/dL and 2.2 mg/dL, respectively (Table 1). The patient was discharged five days after admission with good outcome.

**Table 1:** Progression of parameters during hospital admission in a patient with naphthalene exposure. _ND - not drawn,  R - blood draw refused, * - transfused 4 units packed red blood cells._

	Day 1	Day 2	Day 3 AM	Day 3 PM	Day 4	Day 5
Hemoglobin g/dL	11.0	6.9	R	3.6	*9.6	9.7
Total Bilirubin mg/dL	12.0	11.8	8.0	6.5	5.2	2.2
Creatinine mg/dL	0.3	0.4	0.8	1.4	0.8	0.8
Methemoglobin %	14	ND	ND	10	ND	2
Urine color	Dark brown	Dark brown	Dark brown	Dark brown	Brown	Amber

## Discussion

Naphthalene, a widely used industrial and household chemical, is an uncommon agent of poisoning worldwide, and may be a diagnostic challenge if naphthalene exposure is unknown. Patients initially present with recurrent vomiting, headache, and dark urine [1]. Hemolytic anemia and methemoglobinemia is characteristic of naphthalene poisoning. Severe neutrophilic leukocytosis and acute kidney injury is also observed [4]. Hyperbilirubinemia, with indirect predominance, elevated lactate dehydrogenase and decreased haptoglobin levels are seen [5].

Naphthalene causes oxidative stress by enhancing the production of free oxygen radicals, which then results in lipid peroxidation and deoxyribonucleic acid damage to cells [6]. Hemolysis is thus particularly seen in patients who have a low tolerance to oxidative stress, such as patients with G6PD deficiency [7]. G6PD is essential in red cell metabolism due to its role in the pentose phosphate pathway. It provides resistance for oxidative stresses on the cell, as it leads to the generation of the reduced form of nicotinamide adenine dinucleotide phosphate [8]. The presence of G6PD likely reduces the severity of naphthalene poisoning [5]. The oxidative stress produced by naphthalene results in methemoglobinemia—the oxidized form of hemoglobin. In methemoglobin, the iron (Fe) moiety of unoxygenated hemoglobin is in the ferric (Fe +3) state and does not bind oxygen. The affinity of oxygen for the partially oxidized portion of hemoglobin is thus increased. The patient becomes cyanotic if the methemoglobin level is above 1.5 g/dL. The arterial blood changes to dark brown color [9]. In such patients with methemoglobinemia, pulse oximetry is not reliable. The conventional pulse oximeters use two wavelengths of light, which cannot detect methemoglobin and cannot accurately determine oxygen saturation when methemoglobinemia is present. Pulse oximetry readings in such patients may consistently show an oxygen saturation of 85% despite arterial blood gas oxygen saturation values [10].

Treatment of naphthalene toxicity is supportive, including mechanical ventilation and blood pressure support with the use of inotropes [4]. In mild cases, if the causative agent is removed, the methemoglobin may return to hemoglobin within a few days [9]. However, methemoglobinemia above normal limits can persist 19 days post ingestion of naphthalene-containing products if there is no intervention [5]. Underlying cardiac, pulmonary, or hematologic disease may worsen the toxicity of methemoglobinemia [11]. Ascorbic acid, which acts as a free radical scavenger, can be used to decrease the oxidative stress of naphthalene [12]. Doses of 300 mg by mouth daily have been used in naphthalene poisoning with good outcome [3, 4]. Specific treatment of methemoglobin includes the use of methylene blue. It increases the rate of reduction of methemoglobin to hemoglobin [4]. Recommended doses of methylene blue for the treatment for methemoglobin is 2 mg/kg body weight for infants, 1.5 mg/kg body weight for older children, and 1 mg/kg body weight for adults, in a 1% sterile aqueous solution via slow intravenous infusion [13]. Methylene blue may, however, induce hemolysis and result in paradoxical methemoglobinemia in patients with G6PD deficiency. G6PD testing should be performed prior to administration. N-acetylcysteine (NAC) may also be used in the treatment of methemoglobinemia as a reducing agent, especially in patients with G6PD deficiency [11]. Exchange transfusion is an alternative option in these patients [11].

Management guidelines of naphthalene toxicity are unclear and overall treatment plans may depend on the severity of the clinical picture. Treatment with concentrated red blood cells transfusion and ascorbic acid has resulted in fast normalization of methemoglobinemia [5]. The combination of red blood cell transfusion, intravenous methylene blue, NAC, and ascorbic acid has also been used for the treatment for naphthalene ingestion with good results [1].

## Conclusions

Naphthalene toxicity requires a high level of suspicion if exposure is unknown. It should be suspected in patients with acute onset dark brown urine, nausea, vomiting and diarrhea, combined with acute hemolytic anemia, methemoglobinemia, and acute kidney injury. Exact guidelines for the treatment of naphthalene poisoning based on the mode and severity of toxicity are unknown. Treatment includes supportive care, with intravenous hydration, respiratory and blood pressure support, and possibly renal replacement therapy. Specific management options include ascorbic acid, methylene blue, and NAC. Care should be individualized based on the severity of the clinical picture.
